# Rehabilitation of Cervical Spondylotic Myelopathy and Carotid Space Schwannoma: A Case Report

**DOI:** 10.7759/cureus.67789

**Published:** 2024-08-26

**Authors:** Siddhant S Deshmukh, Pallavi Harjpal

**Affiliations:** 1 Department of Musculoskeletal Physiotherapy, Ravi Nair Physiotherapy College, Datta Meghe Institute of Higher Education and Research, Wardha, IND; 2 Department of Neuro-Physiotherapy, Ravi Nair Physiotherapy College, Datta Meghe Institute of Higher Education and Research, Wardha, IND

**Keywords:** physical therapy rehabilitation, carotid space schwannoma, cervical spondylotic myelopathy (csm), conservative management, neurophysiotherapy

## Abstract

Compression of the spinal cord at the cervical level of the spinal column is the hallmark of the disorder known as cervical myelopathy. The aberrant reflexes, hyperreflexia, pathologic reflexes, clumsiness in the hands and fingers, and disturbance of the gait are caused by this compression. It usually starts slowly, increases gradually, and eventually results in a functional decline. For patients older than 55, the most common cause of spinal cord dysfunction is cervical spondylotic myelopathy (CSM). The traditional definition of the pathogenesis of CSM is multilevel spondylosis, where osteophytes develop as a consequence of disc degeneration. The connection between myelopathy and increasing cervical kyphosis, however, has not received much attention. Myelopathy develops as a result of the kyphosis pushing the spinal cord against the vertebral bodies, causing disease in the anterior cord and increasing longitudinal cord strain because of the cord's tethering by the cervical nerve root and dentate ligaments. Because schwannomas in the carotid area are located close to important neurovascular structures, they can provide particular diagnostic and treatment issues. This case report focuses on the management of a rare condition of CSM and carotid space schwannoma by various neurophysiotherapy approaches over six weeks of rehabilitation.

## Introduction

Compression of the spinal cord at the cervical level of the spinal column is the hallmark of the disorder known as cervical myelopathy. The aberrant reflexes, hyperreflexia, pathologic reflexes, clumsiness in the hands and fingers, and disturbance of the gait are caused by this compression. It usually starts slowly, increases gradually, and eventually results in a functional decline. All the patients having more than 60% canal stenosis (less than 6 mm of disc cord space) will develop myelopathy. The greatest predictor of poor neurologic recovery and perioperative morbidity is age [1]. Pathophysiologically, recurrent dynamic injury resulting from segmental hypermobility, spinal malalignment that modifies cord tension and vascular supply, and static compression of the spinal cord can all lead to symptomatic degenerative cervical myelopathy (DCMs). On the latter, it has been established that unstable spine segments may be responsible for chronic repetitive microtrauma on the spinal cord that is not substantial enough to be recognized as traumatic spinal cord injury (SCI) [2].

For patients who are older than 55, the leading cause of spinal cord dysfunction is cervical spondylotic myelopathy (CSM). The traditional definition of the pathogenesis of CSM is multilevel spondylosis, where osteophytes develop as a consequence of disc degeneration [3]. Static and dynamic mechanical variables are the two groups into which the pathophysiology of cervical spondylotic myelopathy is divided. Disc herniation, congenital spinal canal stenosis, degenerative osteophytosis of the uncovertebral and facet joints, ligamentum flavum hypertrophy and posterior longitudinal ligament, and osteophyte growth in the vertebral bodies are examples of static causes. Aberrant forces applied to the spinal cord and spinal column during cervical spine flexion and extension under normal physiological loads are known as dynamic variables [4].

Overall, cervical myelopathy is detected in 18.1% of patients undergoing cervical decompression myelopathy across the country [5]. Clinical signs of decompressive cervical myelopathy include poor coordination, instability in walking, stiffness or soreness in the neck, clumsiness in the upper extremities, non-dermatomal weakness or numbness, loss of dexterity, weakness in the lower extremities, and urgency in urinating and defecation. The cervical spine's range of motion is assessed during a physical examination. If surgical treatment is recommended, a limited neck extension should be considered to avoid iatrogenic hyperextension injury to the neck [6]. Lhermitte's sign is an electric shock-like sensation that travels down the middle of the patient's back and into the limbs when the neck is flexed, and it may be present in CSM or MS patients [7].

Peripheral nerve myelination is facilitated by Schwann cells, which is the source of schwannoma, which is a benign tumor. Because schwannomas in the carotid area are located close to important neurovascular structures, they can provide particular diagnostic and treatment issues. To achieve thorough resection and preserve nerve function, surgical excision is usually the approach taken in the management of carotid space schwannomas. Although surgical morbidity can be substantial because of the tumor’s proximity to important neurovascular structures, the prognosis is generally good because of the benign nature of the tumor. Physiotherapy is a crucial component of the cervical myelopathy treatment plan, even in cases when schwannomas complicate difficulties. Physiotherapy can greatly improve patient outcomes by combining pain management, strengthening, mobility enhancement, neuromuscular re-education, and posture training. Numerous academic sources attest to the effectiveness of physiotherapy, highlighting its significance in the conservative treatment of cervical myelopathy.

This case report aims to improve the function of the patient by providing rehabilitation and improving his quality of life.

## Case presentation

A 50-year-old female patient was fine until February 2024 when she started experiencing a tingling sensation in the fourth and fifth fingers of her right hand. After a few days, she also started experiencing pain and a tingling sensation in her right foot and complained of a headache. In March, the patient complained of difficulty performing her daily activities in the house, and she visited the hospital for the same. The patient also complained of heaviness in the lower limb, and, because of this, it was difficult for her to walk. Investigations such as an MRI of the brain were advised. The patient is also a known case of carotid space schwannoma, which is the reason why the patient is managed conservatively, and physiotherapy treatment is going on until surgery can be avoided.

Clinical findings

**Figure 1:**
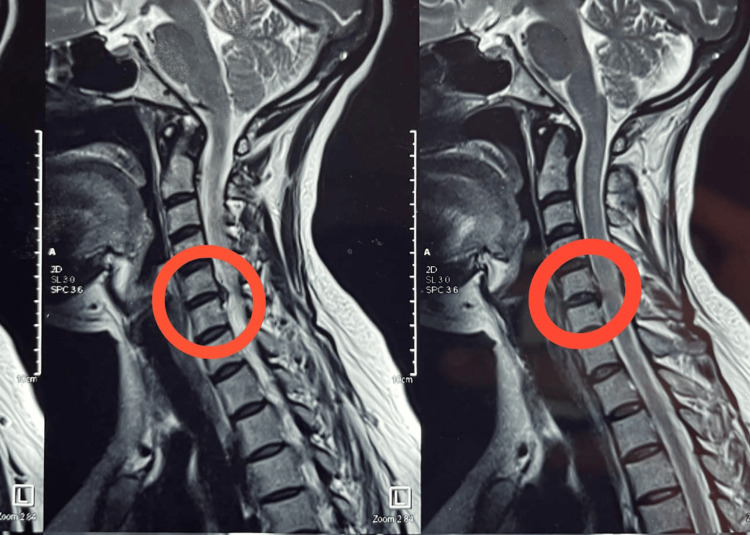
MRI of the cervical spine showing indention to the spinal, which is leading to cervical myelopathy

Before conducting the physical examination, the patient provided verbal and written consent. The patient was observed in a semi-Fowler’s position. All cranial nerves were intact. All the superficial and deep sensations were intact, except pain and temperature in the right hand. The muscle tone of the patient was normal. As mentioned in Table 1, the pre-reflexes of the joint were assessed, which showed normal reflexes, except the Achilles tendon reflex, which was diminished in the lower extremities. Lhermitte’s sign was seen as positive. According to the Oxford grading scale, manual muscle testing (MMT) was taken before treatment (Table 2). The patient was able to sit with minimum assistance. Moreover, the patient was assessed according to the European myelopathy scale (EMS) before the treatment as grade 11. The neurologist advised an MRI of the cervical spine, which revealed compression of the spinal cord at the C4-C5 levels (Figure 1). On clinical examination, the finger-to-nose test was positive, indicating impaired coordination. A foot tapping test was performed to assess the balance of the patient, and it was positive. Sitting balance was assessed using the sitting balance scale, wherein the patient scored 12 out of 44. The patient's balance was assessed using the Berg balance scale and functional independence measure was used as an outcome measure to grade her dependency in performing her daily activities (Table 3).

**Table 1:** Pre-reflexes are taken for both the right and left sides ++: normal reflexes; +: diminished reflexes

Reflexes	Right	Left
Biceps Reflex	++	++
Triceps Reflex	++	++
Brachioradialis Reflex	++	++
Patellar Reflex	++	++
Achilles Reflex	+	+

**Table 2:** Baseline grading of MMT before treatment

Muscle Groups	MMT Grade (Left)	MMT Grade (Right)
Shoulder Flexors	3	3
Shoulder Extensors	3	3
Elbow Flexors	4	4
Elbow Extensors	3	3
Wrist Flexors	3	3
Wrist Extensors	3	3
Hip Flexors	2	2
Hip Extensors	2	2
Knee Flexors	2+	2+
Knee Extensors	2+	2+
Ankle Plantar flexors	2	2
Ankle Dorsi flexors	2	2

**Table 3:** Manual muscle testing after six weeks of physiotherapy intervention

Outcome Measure	After Six Weeks (Left)	After Six Weeks (Right)
Shoulder Flexors	3	3
Shoulder Extensors	3	3
Elbow Flexors	3	3
Elbow Extensors	4	4
Wrist Flexors	4	4
Wrist Extensors	3	3
Hip Flexors	3	3
Hip Extensors	3	3
Knee Flexors	3	3
Ankle Plantar Flexors	3	3
Ankle Dorsiflexors	4	4

Follow-up and outcome measures

The outcome measures of pre- and post-physiotherapy rehabilitation are mentioned in Table 4.

**Table 4:** Outcome measures pre- and post-treatment Grade 1: score ranges from 13-16 points Grade 2: score ranges from 9-12 points

Outcome Measure	At Baseline	After Six Weeks
European Myelopathy Scale (EMS)	11 (Grade 2)	14 (Grade 1)
Berg Balance Scale (BBS)	9/56	22/56
Functional Independence Measure (FIM)	72/126	100/126

Physiotherapy management

After obtaining the referral from the neurologist, physiotherapy was started. The patient had physical therapy rehabilitation six days a week for 30 minutes each day. The protocol to be followed based on a rehabilitation timeframe is presented in Table 5. It was given for six weeks.

**Table 5:** Physiotherapy rehabilitation protocol

Problem	Goal	Intervention	Rationale
Lack of knowledge and awareness of the condition may lead to some complications	To prevent complications	Explain the condition to the patient and the caregiver to prevent complications	To improve the quality of life of a patient
Cervical ROM	To prevent cervical extension	Cervical collar	It supports the head's weight as the bone, soft tissues, and muscles of the neck recover
Decreased coordination	To improve the coordination	Frenkel’s exercises in lying position: (1) moving the heel up to the patella, middle of tibia, and ankle of the contralateral leg; (2) dragging the heel along the tibia. Exercises in sitting position: (1) lifting the thigh with the knee flexed and returning the foot firmly to the ground	To initiate neuromuscular training
Reduced strength	To improve strength	Upper limb (1) shoulder flexors abductors (forward raises, lateral raises); (2) elbow flexors, extensors (biceps curls). Lower limb: (1) hip flexors abductors (hip flexors in sidelying position and abductors in supine position); (2) knee flexors extensors (sidelying position); (3) plantar flexors: dorsiflexors; 4) pnf d1 d2 flexion extension pattern for both upper and lower limbs	Strengthening improves functional performance
Impaired dynamic sitting balance	To improve balance	(1) Sitting with the eyes closed for 30 seconds. (2) Perturbations in all directions in a sitting position. (3) Ball-throwing activity in high sitting	

## Discussion

Nowadays, the most common treatment for cervical myelopathy is surgery, which involves decompressing the cord and supporting the spine [8]. Since the patient discussed is a known case of carotid space schwannoma and managed conservatively. Rhee et al. state that nonoperative care is as effective as surgical treatment for patients with single-level myelopathy, and intramedullary signal change on T2-weighted MRI [9]. The principal aim of neurorehabilitation training is to facilitate gait rehabilitation or recovery, as neurological disorders often result in individuals exhibiting gait abnormalities. These abnormalities encompass muscle weakness, uneven step length, diminished walking speed, an uncoordinated inter-limb walking pattern, compromised balance, and reduced force generation [10]. Kadaňka et al. justified surgical intervention in individuals with compressive cervical myelopathy, and it must be demonstrated that it is superior to the disease's natural progression or conservative care. This is especially true for mild and moderate forms of the disease, which progress slowly or not at all. Most compressive cervical myelopathy patients fall under this category [11]. The FTT is a quick, simple, and cost-effective tool, and it is useful in neurologic examination or a primary care setting. Hayato et al. state that the FTT contributes to the assessment of cervical myelopathy, which is currently underused as part of a routine neurologic examination of compression myelopathy [12].

The patient in the case report has undergone a well-tailored physical therapy rehabilitation protocol made for the patient with exercises prescribed by two qualified orthopedic and neurophysiotherapists. The use of Frenkel’s exercises were used in the treatment to improve coordination through exercises including exercises requiring precision and repetition. Physical therapy is aimed at reducing neurological symptoms and improving the quality of activities of daily living of the patient. Lhermitte’s sign was positive, so a cervical collar was prescribed to avoid aggravating factors. In patients with minimal cord compression in the neutral cervical spine position, appreciable improvement is expected. The goal of the above case study was to shed some light on the management of cervical myelopathy in achieving functional objectives and improving the quality of life of the patient.

## Conclusions

The neurologist opted for conservative management along with neurophysiotherapy. Compared to patients with severe cervical myelopathy, who may require surgical intervention, patients with mild to moderate cervical myelopathy typically develop better from physiotherapy. Early intervention with physiotherapy can lead to better outcomes. Prolonged compression and delayed treatment can result in irreversible damage. The proper adherence to the physiotherapy protocol and active participation by the patient will bring the desired results. Hence, neurophysiotherapy plays a vital role in improving the strength, coordination, balance, and functional independence of these patients.
